# Mapping QTLs for Super-Earliness and Agro-Morphological Traits in RILs Population Derived from Interspecific Crosses between *Pisum sativum* × *P. fulvum*

**DOI:** 10.3390/cimb45010044

**Published:** 2023-01-11

**Authors:** Hatice Sari, Tuba Eker, Hilal Sule Tosun, Nedim Mutlu, Ibrahim Celik, Cengiz Toker

**Affiliations:** 1 Faculty of Agriculture, Department of Field Crops, Akdeniz University, Antalya 07070, Turkey; 2 Department of Crop and Soil Science, Washington State University, Pullman, WA 99164, USA; 3 Faculty of Agriculture, Department of Plant Protection, Akdeniz University, Antalya 07070, Turkey; 4 Faculty of Agriculture, Department of Ag-Biotech, Akdeniz University, Antalya 07070, Turkey; 5 Department of Agricultural and Livestock Production, Pamukkale University, Denizli 20700, Turkey

**Keywords:** *Pisum sativum*, *Pisum fulvum*, earliness, agronomic traits, morphological traits, SSRs, QTLs, recombinant inbred lines (RILs)

## Abstract

Earliness in crop plants has a vital role in prevention of heat-induced drought stress and in combating global warming, which is predicted to exacerbate in the near future. Furthermore, earliness may expand production into northern areas or higher altitudes, having relatively shorter growing season and may also expand arable lands to meet global food demands. The primary objective of the present study was to investigate quantitative trait loci (QTLs) for super-earliness and important agro-morphological traits in a recombinant inbred line (RIL) population derived from an interspecific cross. A population of 114 RILs developed through single-seed descent from an interspecific cross involving *Pisum sativum* L. and *P. fulvum* Sibth. et Sm. was evaluated to identify QTLs for super-earliness and important agro-morphological traits. A genetic map was constructed with 44 SSRs markers representing seven chromosomes with a total length of 262.6 cM. Of the 14 QTLs identified, two were for super-earliness on LG2, one for plant height on LG3, six for number of pods per plant on LG2, LG4, LG5 and LG6, one for number of seeds per pod on LG6, one for pod length on LG4 and three for harvest index on LG3, LG5, and LG6. AA205 and AA372-1 flanking markers for super-earliness QTLs were suggested for marker-assisted selection (MAS) in pea breeding programs due to high heritability of the trait. This is the first study to map QTLs originating from *P. sativum* and *P. fulvum* recently identified species with super-earliness character and the markers (AA205 and AA372-1) linked to QTLs were valuable molecular tools for pea breeding.

## 1. Introduction

The *Pisum* L. genus is classified in the Fabaceae (Legumes) family, Fabaoideae (Papilionoideae) sub-family and Fabeae Rchb. tribe. The genus *Pisum* consists of three cultivated species including *P. sativum* L. (garden pea), *P. arvense* (L.) Poir. (field pea) and *P. abyssinicum* A.Br. (Dekoko or Abyssinian/Ethiopian pea) and there are seven taxa in the genus. These taxa were classified as follows: *P. sativum* L subsp. *sativum* var. *sativum*, *P. sativum* subsp. *sativum* var. *arvense* and *P. sativum* subsp. *abyssinicum* as cultivated species, while *P. elatius* (M.Bieb.) Asch. and Graebn. complex contains three varieties including *P. sativum* subsp. *elatius* var. *elatius, P. sativum* subsp. *elatius* var. *pumilio* Meikle and *P. sativum* subsp. *elatius* var. *brevipedinculatum* Davis and Meikle [1]. *P. fulvum* Sibth. et Sm. with a small distribution in Middle East and Turkey is the most distinct relative of the garden pea [2,3].

Pea has a central place in the history of genetics as an experimental plant since Mendel studied the famous laws of heredity [4]. Garden pea is among the most important food legumes, fodders and vegetable crops. It is grown in 99 countries worldwide. World annual production quantity of garden pea was reported to be 14.6 million tons for dry pea and 19.9 million tons for vegetable pea in 2020 [5]. It is used for various purposes, including food (leaves, green pods, unripe fresh seeds and dry mature seeds) and feed (direct grazing and silage). Garden pea is quite rich in protein (21–33%), starch (37–49%), soluble sugars (5%), fiber (2–9%), minerals and vitamins [2,6,7].

Garden pea, like faba bean (*Vicia faba* L.), is a cool season food legume and more susceptible to droughts than chickpea (*Cicer arietinum* L.), lentil (*Lens culinaris* Medik.) and grass pea (*Lathyrus sativus* L.) [8]. Like other crop plants, earliness provides many advantages in garden pea cultivation, such as prevention from drought-induced heat stress. Three resistance mechanisms have been reported for heat and drought: (i) escape, (ii) avoidance and (iii) tolerance. Escape is provided by early phenology including earliness [9,10]. Drought-induced heat stress has already increased due to climate change and is predicted to worsen due to a rise in temperature up to 1.5–4 °C in the near future [11]. Breeding for heat-tolerant garden pea has crucial importance with early flowered and matured cultivars [12]. Success in the selection for heat-tolerant garden pea depends on accuracy of selection with few efforts and short times by marker-assisted selection (MAS) [13,14]. New genetic sources for earliness, yield and yield-related traits should therefore be studied and mapped in garden pea.

Pea has a quite large genome of about 4.45 Gb [15]. Thanks to high-resolution genetic maps, it is possible to identify genes or QTLs controlling important agro-morphological and desirable traits. QTL mapping studies were performed in pea for many characteristics using maps constructed with molecular markers [16,17,18,19,20,21,22,23,24,25,26,27,28,29,30]. Dirlewanger et al. [16] identified a QTL for earliness on chromosome 6 (LG2) in the F_2_ population of pea. Prioul et al. [19] discovered three QTLs for days to flowering on LGs 2, 3 and 6 in RILs, derived from intraspecific crosses. Timmerman-Vaughan et al. [20] identified four QTLs for days to flowering on LGs 1, 2b, 3 and 5b in A26 × Rovar population and three QTLs on LGs 1, 3 and 5b in A88 × Rovar population. Fondevilla et al. [23] detected four QTLs for earliness on LG2, LG3, and LG6 in RILs, derived from the cross between *P. sativum* subsp. *syriacum* and *P. sativum*. Furthermore, four QTLs were determined for days to flowering on LGs 3, 4 and 5 in interspecific crosses between *P. sativum* and *P. fulvum* by Jha et al. [28]. Huang et al. [30] identified three QTLs for days to flowering on LGs 2, 3 and 6b in intraspecific crosses.

There are also important QTL studies on pea seed quality. QTLs were determined in Va and Vb of the linkage map for seed protein content [31]. In addition to important minerals such as Ca, Fe, K, Mg, Mn, Mo, and P [32], QTLs of starch, fiber, and phytate contents [33] in pea were also defined. Various QTL mapping studies have been performed for agro-morphological traits of pea. The majority of QTLs for plant height were determined on LG3 [18,19,28,33,34,35,36,37]. Furthermore, several QTLs were identified for morphological traits, like number of pods per plant, number of seeds per pod, pod size, biological yield, seed yield and harvest index on all seven linkage groups [31,38,39,40,41]. However, because quantitative traits are polygenic and influenced by environmental conditions, it may be scattered in linkage groups. The QTL studies using plant populations derived from interspecific crosses in pea are very limited [28,42].

Promising interspecific crosses of pea were previously reported to have a potential for further improvement of super-earliness [43]. Thus, the objectives of this study were to: (i) generate a genetic linkage map of a pea recombinant inbred line (RIL) population derived from a cross between *P. sativum* (♀) and *P. fulvum* (♂) × (ii) discover novel QTLs associated with super-earliness and important agro-morphological traits.

## 2. Materials and Methods

### 2.1. Plant Material

A total of 114 F_4_ recombinant inbred lines (RILs) derived from the *P. sativum* × *P. fulvum* interspecific crosses were used as the plant material of the present study. The parents were selected based on contrasting or significant differences in morphological and phenological characteristics. The female parent, ACP 20 (*P. sativum*), is a large, wrinkled and cream color-seeded and early-flowered genotype, while the male parent, AWP 600 (*P. fulvum*), is a small, smooth and black color-seeded and late-flowered genotype [43].

ACP 20 is a landrace from Antalya, Turkey, whereas AWP 600 originated in Turkey and was obtained from USDA GRIN in the United States. Each recombinant inbred line was advanced as five seeds after the F_2_ population. That is, from F_3_ to F_4_, each line was advanced as a family consisting of five individuals. Both parents and RILs were evaluated in the years 2019 and 2020 under glasshouse conditions.

### 2.2. Phenotyping

Phenotyping for QTL was recorded on 12 characteristics, namely as flower color (FC), days to flowering (DF; days), days to pod setting (DP; days), plant height (PH; cm), first pod height (FH; cm), internode length (IN; cm), number of pods per plant (PP), number of seeds per pod (SP), pod length (PL; cm), biological yield per plant (BY; g), seed yield per plant (SY; g) and harvest index per plant (HI; %). Phenotyping was recorded in parents and F_4_ lines derived from interspecific crosses *P. sativum* × *P. fulvum*. Phenotyping was evaluated by averaging the five families grown on F_4_ lines for each characteristic. DF was recorded as the number of days after germination until the first flowering. DP was recorded as the number of days after germination until the first pod setting. PH and FH were recorded in cm as the height of a plant from the ground to the top of the plant and as the height from ground to the first pod, respectively. Internode length (IN) was measured at the distance between two stipules with a ruler. PP and SP were recorded as the total number of pods per plant and seeds per pod, respectively. PL was recorded in cm as the length of a pod. BY was recorded in grams (g) as the total weight of a plant after harvest, while SY was recorded in g as the weight of seeds per plant after harvest. HI was calculated in percentage (%), as the ratio of SY to BY multiplied by 100. For PL, three randomly selected pods of each plant were used and SP of the same pods was recorded.

### 2.3. Genotyping

DNA isolation was carried out according to the CTAB method developed by Doyle and Doyle [44] using young leaves. In order to create the genetic map and determine the QTLs, a total of 70 SSR markers, 10 from each linkage group (LG), were selected from the SSR markers mapped by Loridon et al. [21]. Forty-five SSRs markers showing polymorphism in both the female and the male parent were used in this study. Some information about polymorphic markers is presented in Table 1.

The polymerase chain reaction (PCR) mix was prepared in a total volume of 15.47 µL, 1.5 µL dNTP, 1.25 µL MgCl2, 1.5 µL PCR buffer, 0.1 µL Taq DNA polymerase, 7.62 µL ultrapure water, 1 µL F primer, 1 µL R primer and 1.5 µL DNA for each sample. In PCR amplification, initial denaturation at 95 °C for 3 min, then at 94 °C for 50 s, at 45–55 °C for 40 s, at 72 °C for 50 s, final extension after 35 cycles at 72 °C for 5 min were completed. After the PCR amplification was completed, 3% agarose gel electrophoresis was used to visually examine and score the separation of the formed bands based on the size differences of the obtained PCR products. A 1 kB plus marker (Thermo Scientific GeneRuler 1kB plus DNA Ladder) was used to determine product sizes (molecular weights). The electrophoresis tank, which is connected to the power source, was run at 75 volts for about 100 min and the bands were separated from each other.

Of the F_4_ RILs, those with the same band size as the female parent were scored as “A”, those with the same band size as the male parent as “B” and those with both parent bands were scored as “H”. Lines that did not show bands were scored as “-”.

### 2.4. Genetic Mapping

The genetic linkage map was created using the Join-Map 4.1 software [45]. Markers were assigned to linkage groups (LGs) with a LOD greater than 3 using the Kosambi map method of Join-Map 4.1. The linkage groups identified in this study were aligned to seven pea chromosomes based on common markers in the pea genetic map previously reported [21].

### 2.5. QTL Analyses

The QGene software was used for QTL analysis of days to flowering, days to pod setting, plant height, first pod height, internodes, number of pods per plant, number of seeds per pod, pod length, biological yield, seed yield and harvest index. Using the Composite Interval Mapping (CIM) method, the QTL was determined for each quantitative trait with LOD > 3. MapChart 2.0 [46] software was used to mark the determined QTLs on the created genetic map.

Gene action was calculated by dividing the absolute value of the estimated dominance effect (|d|) by the absolute value of the estimated additive effect (|a|) [47]. |d|/|a| = 0–0.20 additive (A); partial dominance (PD) between 0.21–0.80; dominance (D) between 0.81–1.20; and >1.20 have been classified as over-dominance (OD).

## 3. Results

### 3.1. Phenotypic Characteristics

**Figure 1:**
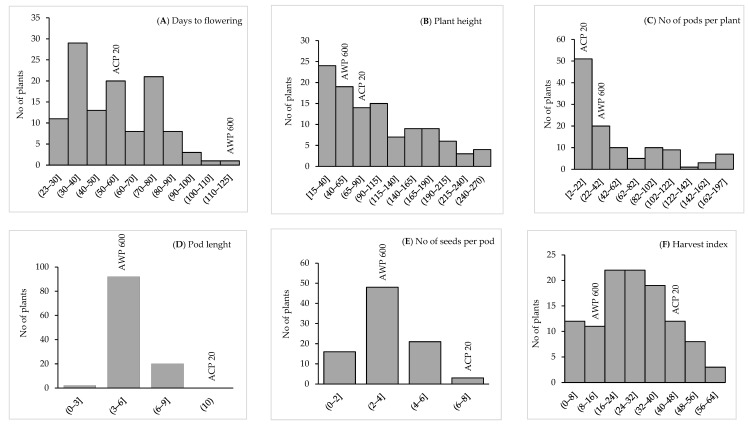
Frequency distribution of (**A**) days to flowering, (**B**) plant height, (**C**) no of pods per plant, (**D**) pod length, (**E**) no of seeds per pod, and (**F**) harvest index in the ACP 20 (*P. sativum*), AWP 600 (*P. fulvum*) and F_4_ population derived from interspecific crosses between *P. sativum* × *P. fulvum*.

Days to flowering was recorded as 53 days for *P. sativum* and 117 days for *P. fulvum*, while the earliest lines flowered 24 days after germination in the F_4_ population. Days to pod setting was 62 days for *P. sativum* and 128 days for *P. fulvum*. On the other hand, the earliest lines formed pods in 30 days in the F_4_ population (Table 2). Plant height for *P. sativum* and *P. fulvum* was 90 and 41 cm, respectively, whereas it varied between 15 to 266.7 cm for the F_4_ population. The number of pods per plant was 11 for *P. sativum* and 23 for *P. fulvum*, ranging between 2 to 197 in the F_4_ population. Pod length was 10 cm for *P. sativum* and 4 cm for *P. fulvum*, while it was between 3 to 9 cm in F_4_ lines. The number of seeds per pod in *P. sativum* and *P. fulvum* was seven and three, respectively, and it was between one to seven in F_4_ lines. The harvest index was 47% for *P. sativum* and 15% for *P. fulvum*, whereas it ranged from 1% to 58% in F_4_ lines (Table 2). Distributions of parents and lines for each characteristic are presented in Figure 1.

### 3.2. Genetic Mapping

**Figure 2:**
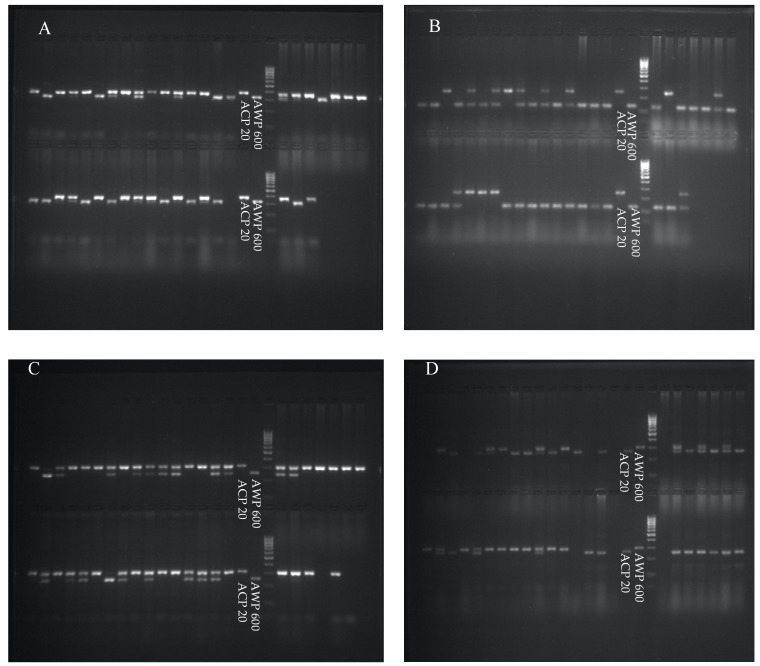
Agarose gel images of markers (**A**) AA67, (**B**) AD148, (**C**) AD270, and (**D**) AA285 between parents and F_4_ lines derived from interspecific crosses involving *P. sativum* × *P. fulvum*.

A population of 114 F_4_ RILs obtained from *P. sativum* × *P. fulvum* interspecific crosses was screened with 70 codominant SSR markers. Of these 70 SSRs, 45 of them showed polymorphism between parents (Figure 2). After each marker was scored on the population, the markers with a LOD greater than three were selected and a genetic linkage map was generated, resulting seven linkage groups. The 44 SSR markers were mapped on LGs. The total length of the map is 262.6 cM, with an average marker resolution of 5.9 cM (Table 3). The number, names and resolution of the SSR markers for each LG are given in Table 3.

### 3.3. QTLs Analyses

**Figure 3:**
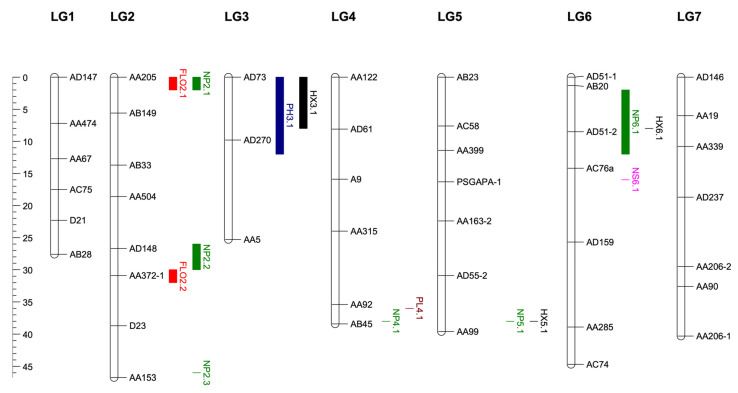
QTLs mapping for FLO: days to flowering, PH: plant height, NP: number of pods per plant, NS: number of seeds per pod, PL: pod length, HX: harvest index in 114 RILs developed from interspecific crosses between *P. sativum* and *P. fulvum*.

A total of 14 QTLs on five different linkage groups were determined for earliness and important agro-morphological traits. Two QTLs were determined on LG2 for flowering time. The first QTL FLO2.1 had a LOD value of 3.6. The AA205 marker, which explained 14% of the phenotypic variance, was the closest to the QTL (Table 4). The second QTL FLO2.2 explained 14% of the phenotypic variance with a LOD of 3.2. The marker AA372.1 was the closest to the QTL (Figure 3). FLO2.1 and FLO2.2 showed a dominance/additive (d/a) ratio of 1.06 and 0.71, indicating a dominant and partially dominant gene action, respectively (Table 4). The QTLs for flowering time explained 28% of the total phenotypic variance.

One QTL for plant height was identified on LG3 with a LOD value of 4.35. Two flanking markers were determined for PH3.1 QTL (Figure 3). The QTL flanked by markers AD73 and AD270 explained 16% of phenotypic variation. PH3.1 showed d/a ratio of 1.65, indicating an over-dominance gene action (Table 4).

Six QTLs were determined for the number of pods per plant, one of the most important yield components. Three QTLs were mapped on LG2, one on LG4, one on LG5 and one on LG6. The NP2.1, NP2.2 and NP2.3 on LG2 were mapped with LOD values of 4.3, 3.5 and 3.6, respectively (Figure 3, Table 4). The NP2.1 and NP2.2 explained 16% and 13% of the phenotypic variation, respectively.

The NP2.3, NP4.1 and NP5.1 explained 13%, 14% and 14% of the variation (Table 4, Figure 3). The NP6.1 QTL associated with the number of pods per plant explained 17% of the phenotypic variation (Table 4). The six QTL determined for the number of pods per plant within the scope of this study explained a total of 87% of the variation. NP2.1, NP2.2, NP5.1 and NP 6.1 had over dominance gene action. The HX3.1 QTL associated with the harvest index explained 13% of the phenotypic variation (Table 4). The second QTL (HX5.1) explained 12% of the phenotypic variation for the same trait. The third QTL (HX6.1) on LG6 explained 12% of the phenotypic variation. The three QTLs for harvest index explained 37% of the total phenotypic variation. The PL4.1 QTL associated with the pod length explained 12% of the phenotypic variation (Figure 3, Table 4). The NS6.1 associated with the number of seeds per pod explained 13% of the phenotypic variation (Table 4).

## 4. Discussion

A total of 114 F_4_ RILs derived from *P. sativum* × *P. fulvum* interspecific crosses were used for phenotyping and genotyping. The 70 SSR markers were selected from the genetic map created by Loridon et al. [21]. The 45 SSRs showed parental polymorphisms. The linkage map with seven linkage groups was created using 44 SSRs with a LOD greater than 3.0 using the Kosambi function. Each LG represents a pea chromosome and the total map length was 262.6 cM with an average marker resolution of 5.9 cM (Table 3). The pea genetic map with the highest number of SSR markers was reported by Loridon et al. [21] with 239 polymorphic markers. In this study, a genetic linkage map was created by using of the RIL population developed from interspecific crossing. Common markers used in both studies are indicative of cross-population transferability.

Days to flowering, days to pod setting, plant height, first pod height, internode, number of pods per plant, number of seeds per pod, pod length, biological yield, seed yield and harvest index were evaluated to determine QTLs in this study. The evaluated characteristics are important targets for pea breeding. Of these characteristics, 14 QTLs were determined for a total of six traits: days to flowering, plant height, number of pods per plant, number of seeds per pod, pod length and harvest index. In the QTL analyses, composite interval mapping (CIM) was used. The F_4_ RIL population of 114 individuals derived from *P. sativum* × *P. fulvum* interspecific crosses was used to determine the QTLs. In a study comparing RIL populations for QTL detection, it was concluded that the F_4_ RIL population may be as effective as the F_6-7_ populations [48].

Flowering time is one of the main determinants of adaptation to different ecological and geographical regions. Early-flowering genotypes in pea play an important role in minimizing bottlenecks such as abiotic and biotic stresses. There are growing global concerns about the impact of climate change on food production, livelihoods and food security [49,50]. Global warming is thought to harm agricultural production and is one of the most serious threats to food supply. The second threat is the increasing world population, estimated at 8 billion by 2030, which will require a 60% increase in current food production [51,52]. The majority of the world’s population lives in cities, and considering the reasons for migration from rural areas to cities, it is inevitable that the consumption rate will create even more food deficits [50]. According to the data of the International Panel on Climate Change (IPCC), global warming will exceed 1.5 °C by 2030, causing permanent loss of the most sensitive ecosystems. It is thought to cause a crisis for societies in underdeveloped and developing countries. Super-early individuals from the previous study could escape high temperature stress, while late-maturing individuals were exposed to heat stress during the flowering and pod setting periods [43]. The earliest lines in the F_4_ population flowered 24 days after germination, while *P. sativum* flowered in 53 days and *P. fulvum* flowered in 117 days (Table 2). *P. sativum* required 62 days to reach pod setting, while *P. fulvum* required 128 days. In the F_4_ population, the earliest lines developed pods in 30 days (Figure 1). In a previous study, the earliest days to flowering in the F_2_ and F_3_ generations of the same population were 17 and 13 days under short-days, respectively [43].

More than 20 loci related to flowering time and flowering development had been identified in pea and the interactions of these loci determined flowering time. Late-flowering (*Lf*) [53], high-response (*Hr*), sterile nodes (*Sn*), early (*E*), photoperiod (*Ppd*) [54] and die Neutralis (*Dne*) loci are the most important ones [55,56,57]. The *Ppd* and *Lf* loci were mapped on LG2 [54,56], while the *Hr* and *Dne* loci were mapped on LG3 [58,59]. In this study, two QTLs associated with the markers AA205 and AA372.1 were found for flowering time, which are in the same linkage group (on LG2) as the *Ppd* and *Lf* loci. Guindon et al. [40] reported that seed diameter and seed weight characteristics were associated with the AA205 marker in peas. Three genomic regions controlling flowering time were identified on LG2, LG3, and LG6 by Prioul et al. [19]. QTL *flo1* was mapped on LG2, the same linkage group as the QTL found in this study, contributing most of the variation [19]. QTL determined on LG2 was associated with the AB33 marker. The marker flanking the AB33 marker was AA372.1 and it was linked with the *FLO2.2* determined in this study (Table 3). In addition, QTL *flo2* was mapped on LG3 and QTL *mpIII-3* was reported to be in the same region with the pea blight resistance QTL. Resistance alleles in the blight resistance-related QTLs had been associated with alleles that delay flowering time [19]. Burstin et al. [60] mapped one QTL in 49 cM of LGV where the *Det* gene is located for flowering time. Foucher et al. [61] reported that the *Det* gene played a role in the regulation of flowering time. Fondevilla et al. [62] determined two QTLs on LG3 for earliness in pea. In addition, it was reported that the QTLs were close to the AB64 and AA175 markers. QTL was mapped for earliness in pea on LG2 by Dirlewanger et al. [16]. Jha et al. [28] identified four QTLs for flowering time at LGs 3, 4 and 5. In a recent study, three QTLs, two on LG1 and one on LG2, were mapped for flowering time in F_2_ and F_3_ populations obtained from DDR14 and Explorer intraspecific crosses [40]. Fondevilla et al. [23] defined QTLs for flowering time on LG6 and LG3. QTL on LG3 determined by Fondevilla et al. [23] was reported to be related to earliness in the study by Timmerman-Vaughan et al. [20]. Although QTLs determined for flowering time in the previous studies were close to AA205 and AA372.1 markers, it was not directly related. QTL studies on flowering time in peas are limited and two more new QTLs were found on LG2 with this study.

Major and minor QTLs have been identified for plant height in peas in previous studies. Tar’an et al. [18] determined three main QTLs with a total variation of 64.6% and Hamon et al. [34] identified three minor QTLs on LG3. Three QTLs were determined for plant height in LG2, LG3, and LG7 by Prioul et al. [19] and it was emphasized that the QTL on LG3 explained 63% of the variation. Gali et al. [36] identified a major QTL for plant height on LG3, explaining 33–65% of the phenotypic variance in the three RIL populations. Also, Ferrari et al. [35] mapped QTL for plant height on LG3. Gali et al. [33] identified four loci on LG3 associated with plant height using the GWAS (genome-wide association) method. Guindon et al. [40] found QTL on LG2 for plant height. In one study, two QTLs were found, one QTL on LG3 and one QTL on LG5 [37]. In Jha et al. [28], in which quantitative loci of blight disease in pea were studied, five QTLs associated with plant height were identified. Three of the QTLs were positioned on LG3, LG4 and LG7. Although QTLs were found in different linkage groups related to plant height in previous studies, the majority of QTLs that explain the phenotypic variation were identified on LG3, as reported in this study.

Previous studies using RIL populations have identified multiple QTLs associated with the number of pods per plant in more than one linkage group. For the number of pods, a total of five QTLs were determined on LG1, LG2, LG3, LG5 and LG6 [38]. Guindon et al. [40] detected a QTL in the LG1 for the same trait. Sadras et al. [41] determined the QTL in LG2 for the number of pods per m^2^. QTL was determined for the number of seeds per pod on LG2 in an RIL population [31]. Two QTLs were identified for the number of seeds per pod on LG1 by Guindon et al. [40]. Timmerman-Vaughan et al. [39] determined a total of seven QTLs on LG1, LG2, LG3, LG4 and LG7 linkage groups for the number of seeds per m^2^. Sadras et al. [41] found QTL on LG2 and LG3 for the number of seeds per pod. In addition, it was reported that the QTLs of flowering time and yield components were mostly on LG2 [41]. Four QTLs were identified, explaining 40% of the total phenotypic variation for number of seeds per plant by Timmerman-Vaughan et al. [39]. Two of these QTLs were mapped on LG3, one on LG1 and one on LG2 [39]. A QTL (*PL4.1*) associated with the pod length was detected in LG4 with a LOD value of 3.08 (Table 1). It explained 12% of the phenotypic variation. The closest marker to the PL4.1 QTL was AA92 and its position on the map was 36 cM (Figure 3). One QTL for pod size was mapped on LG2 [40].

Another QTL mapped in this study was the harvest index. The *HX3.1* QTL explained 13%, and *HX5.1* and *HX6.1* QTL each explained 12% of the phenotypic variation. The three QTLs explained a total of 37% of the phenotypic variation (Table 4). In a similar study, four QTLs were identified that explained a total of 40% of phenotypic variation. Two of these were mapped on LG3, one on LG1 and one on LG2 [39]. Yield components can be included in many linkage groups due to the characteristics that are easily affected by the environment and the populations.

QTL studies were carried out in pea for various characteristics. In addition to physiological traits, yield and yield components, QTL studies were also conducted against biotic and abiotic stress factors. In a study investigating the relationship between lodging resistance and plant height in pea, lodging resistance was mapped on LG3 [18], and in pea blight disease resistance on LG2, LG3, LG5 and LG6 [19,28], seed color, grain weight, grain yield, biological yield, protein content, broomrape resistance and powdery mildew resistance QTLs were reported in all seven linkage groups for many characteristics [22,63,64,65,66]. Studying QTLs mapped in the same linkage group in future studies may strengthen the functionality of the markers used.

The super-earliness character has a high heritability in this interspecific population [43] and thus MAS can be employed to transfer it into cultivated types.

In conclusion, a 262.6 cM long genetic map was constructed with 44 SSRs markers. A total of 14 QTLs were mapped, two QTLs for super-earliness on LG2, one for plant height on LG3, six QTLs for number of pods per plant on LG2, LG4, LG5 and LG6, one for number of seeds per pod on LG6, one for pod length on LG4 and three for harvest index on LG3, LG5, and LG6. The SSR markers AA205 and AA372-1 flanking super-earliness QTLs can potentially contribute significantly to future marker-assisted pea breeding programs.

## Figures and Tables

**Table 1:** List of SSRs primers used for this study with primer sequences.

Primers	Linkage Groups	Forward (5′-3′)	Reverse (3′-5′)
AD147	LG1	AGCCCAAGTTTCTTCTGAATCC	AAATTCGCAGAGCGTTTGTTAC
AA474	LG1	GCCCACACAAGTGGTTCTATAAAT	ATTAGTCGTTTTTCTGAAACATCAAAG
AA67	LG1	CCCATGTGAAATTCTCTTGAAGA	GCATTTCACTTGATGAAATTTCG
AC75	LG1	CGCTCACCAAATGTAGATGATAA	TCATGCATCAATGAAAGTGATAAA
D21	LG1	TATTCTCCTCCAAAATTTCCTT	GTCAAAATTAGCCAAATTCCTC
AB28	LG1	CCTGAGTCATCACATAGGAGAT	GCAGAAGTATTTGACTTGATGGAA
AA205	LG2	TACGCAATCATAGAGTTTGGAA	AATCAAGTCAATGAAACAAGCA
AB149	LG2	ACAAAGGATGATGAAAGACCCG	TCATTACTCAAAGAATGCACCCAC
AB33	LG2	CATTGAATTTGTGGGAGAAAGG	TGTGGATGTTGCAATTTCGT
AA504	LG2	TGAGTGCAGTTGCAATTTCG	TCAGATGAAGAGCATGTGGG
AD148	LG2	GAAACATCATTGTGTCTTCTTG	TTCCATCACTTGATTGATAAAC
AA372.1	LG2	GAGTGACCAAAGTTTTGTGAA	CCTTGAACCCATTTTTAAGAGT
D23	LG2	ATGGTTGTCCCAGGATAGATAA	GAAAACATTGGAGAGTGGAGTA
AA153	LG2	TTTGATAGTCCGACTTTTCCAT	GTGACAAAAGAATTCAAAACGC
AD73	LG3	CAGCTGGATTCAATCATTGGTG	ATGAGTAATCCGACGATGCCTT
AD270	LG3	CTCATCTGATGCGTTGGATTAG	AGGTTGGATTTGTTGTTTGTTG
AA5	LG3	TGCCAATCCTGAGGTATTAACACC	CATTTTTGCAGTTGCAATTTCGT
AA122	LG4	GGGTCTGCATAAGTAGAAGCCA	AAGGTGTTTCCCCTAGACATCA
AD61	LG4	CTCATTCAATGATGATAATCCTA	ATGAGGTACTTGTGTGAGATAAA
A9	LG4	GTGCAGAAGCATTTGTTCAGAT	CCCACATATATTTGGTTGGTCA
AA315	LG4	AGTGGGAAGTAAAAGGTGTAG	TTTCACTAGATGATATTTCGTT
AA92	LG4	AAGGTCTGAAGCTGAACCTGAAGG	GCAGCCCACAGAAGTGCTTCAA
AB45	LG4	ATTACACCAACAATCTCCCACT	TGTAGAAGCATTTGGGTAGTTG
AB23	LG5	TCAGCCTTTATCCTCCGAACTA	GAACCCTTGTGCAGAAGCATTA
AC58	LG5	TCCGCAATTTGGTAACACTG	CGTCCATTTCTTTTATGCTGAG
AA399	LG5	CCATTGGTATATGAAAGATCGCT	TCCCAATTAATATGGCTAGGCT
PSGAPA-1	LG5	GACATTGTTGCCAATAACTGG	GGTTCTGTTCTCAATACAAG
AA163.2	LG5	TAGTTTCCAATTCAATCGACCA	AGTGTATTGTAAATGCACAAGGG
AD55.2	LG5	AACACATTAACTAAGTCCACAC	AAACCTATCACTTTAGAAACCT
AA99	LG5	AACAATAACATGGCAAAGATT	ACCTTGCGATATAATTGATG
AD51	LG6	ATGAAGTAGGCATAGCGAAGAT	GATTAAATAAAGTTCGATGGCG
AB20	LG6	TTGCATCCCACACAAGTGGT	ACCTCCAGGTTCTGCCTTATCT
AC76a	LG6	CCCAATCCAATAAATAAAGAAA	AATGGTTGTTATGCCATTTT
AD159	LG6	AGCTTGGAACCACAAGATTAGT	GTGAATGATAATTCTCACCCTC
AA285	LG6	TCGCCTAATCTAGATGAGAATA	CTTAACATTTTAGGTCTTGGAG
AC74	LG6	CCTTAGTGTTCTTCAACTC	ACAGAACCAAGTTATCAATA
AD146	LG7	TGCTCAAGTCAATATATGAAGA	CAAGCAAATAGTTGTTTTGTTA
AA19	LG7	GCAGTTGTTGTACCCTAAAATT	TGTATTAGATGAAATTTTGTTTCTC
AA339	LG7	GTGTAGAAGTATTTTACTTGATG	CATCTATTGAAGGAAAATTAT
AD237	LG7	AGATCATTTGGTGTCATCAGTG	TGTTTAATACAACGTGCTCCTC
AA206	LG7	CTGAGAACTCAACGCTCAGACG	CGAGGGTCGAGTTCTGAGATTT
AA90	LG7	CCCTTACCATATTTCGTTTCT	TGCGACTCCATTCTAGTATTG

**Table 2:** Minimum (Min) and maximum (Max) values, means ($\bar{X\;}$ ) and ± standard errors ($S_{\bar{X}}$ ) for phenological and morphological traits in parents and F_4_ population originated from interspecific crosses *P. sativum* × *P. fulvum*.

Traits	ACP 20		AWP 600		F_4_	
	Min-Max	$\bar{X\;}\pm S_{\bar{X}}$	Min-Max	$\bar{X\;}\pm S_{\bar{X}}$	Min-Max	$\bar{X\;}\pm S_{\bar{X}}$
Days to flowering	50–56	53.1 ± 0.7	115–119	116.7 ± 0.7	24–103	54.3 ± 1.8
Days to pod setting	59–63	61.7 ± 0.4	127–130	128.3 ± 0.6	30–114	63.1 ± 1.9
Plant height (cm)	62–117	89.8 ± 8.1	39–43	41 ± 0.7	15–266.7	98.8 ± 6.1
First pod height (cm)	17–40	27.8 ± 3.9	5–6	5.3 ± 0.2	2–75.4	14.9 ± 1.4
No of Internodes	8–9	8.8 ± 0.2	2–3	2.5 ± 0.2	1.6–11.7	5.5 ± 0.2
No of pods per plant	4–21	11.3 ± 2.5	21–24	22.8 ± 0.5	2–197	44.4 ± 4.8
No of seeds per pod	5–8	6.7 ± 0.4	2–3	2.7 ± 0.2	1–7	3.1 ± 0.1
Pod length (cm)	9–11	10.3 ± 0.3	4–4.5	4.2 ± 0.1	2.8–9	5 ± 0.1
Biological yield (g/plant)	16.4–265.6	96.4 ± 38.7	26.7–28.7	27.5 ± 0.3	2–371.1	91.5 ± 8.6
Seed yield (g/plant)	9.6–102.5	40.9 ± 14.8	3.7–4.3	4 ± 0.1	0.4–94.9	20.7 ± 2.2
Harvest index	35.2–58.3	47 ± 3.7	13.9–15.6	14.6 ± 0.3	0.6–58.4	27.3 ± 1.4

**Table 3:** Linkage group (LG), number of SSR markers, length (cM) and average marker distance (cM) of the linkage groups developed with 44 SSRs using F_4_ lines involving *P. sativum* × *P. fulvum*.

LGs	Map Length (cM)	No. of Markers	Resolution of Markers (cM)
LG1	27.6 cM	6	4.6 cM
LG2	46.7 cM	8	5.8 cM
LG3	25.3 cM	3	8.4 cM
LG4	38.4 cM	6	6.4 cM
LG5	39.6 cM	7	5.6 cM
LG6	44.7 cM	7	6.3 cM
LG7	40.3 cM	7	5.7 cM
Total	262.6 cM	44	5.9 cM

**Table 4:** QTLs detected for days to flowering, plant height, no. of pods per plant, no. of seeds per pod, pod length and harvest index under glasshouse conditions.

Characters	QTL	Linkage Group (LG)	Map Position (cM)	R^2^ *	Flanking Markers	Genetic Effect		Gene Action ***
						Add **	Dom	|d|/|a|
Days to flowering	*FLO2.1*	LG2	0–2 cM	0.13	AA205	6.5	−6.9	1.06 D
	*FLO2.2*	LG2	30–32 cM	0.13	AA372.1	6.5	−4.6	0.71 PD
Plant height	*PH3.1*	LG3	0–12 cM	0.16	AD73; AD270	−28.6	47.2	1.65 OD
Pods per plant	*NP2.1*	LG2	0–2 cM	0.16	AA205	10.4	−17.9	1.72 OD
	*NP2.2*	LG2	26–30 cM	0.13	AD148	10.6	−24.3	2.29 OD
	*NP2.3*	LG2	46–46 cM	0.13	AA153	11.0	−3.3	0.30 PD
	*NP4.1*	LG4	38–38 cM	0.14	AB45	12.7	11.2	0.88 D
	*NP5.1*	LG5	38–38 cM	0.14	AA99	10.0	−23.1	2.31 OD
	*NP6.1*	LG6	2–12 cM	0.17	AB20; AD51-2	9.2	463.5	50.38 OD
Seeds per pod	*NS6.1*	LG6	16–16 cM	0.13	AC76a	1.8	19.8	11.0 OD
Pod length	*PL4.1*	LG4	36–36 cM	0.12	AA92	0.42	0.47	1.1 D
Harvest index	*HX3.1*	LG3	0–8 cM	0.13	AD73	8.0	−3.2	0.39 PD
	*HX5.1*	LG5	38–38 cM	0.12	AA99	3.6	−15.8	4.38 OD
	*HX6.1*	LG6	8–8 cM	0.12	AD51-2	4.0	−300.2	75.0 OD

* R^2^ is the percentage of phenotypic variation individually explained by each QTL; ** A negative sign reflects that the QTL alleles which increased were contributed by the wild parent, whereas a positive value means that alleles were donated by the cultivated parent; *** Gene action shows dominance (D), partial dominance (PD) or over-dominance (OD).

## Data Availability

All data are available in this article.
